# Microstructural modelling based on diffusion weighted imaging to guide dose painting in carbon ions for large sacral chordomas

**DOI:** 10.1016/j.phro.2025.100887

**Published:** 2025-12-07

**Authors:** Giovanni Parrella, Letizia Morelli, Giuseppe Magro, Lars Glimelius, Jakob Ödén, Mario Ciocca, Sara Imparato, Marco Rotondi, Maria Rosaria Fiore, Ester Orlandi, Guido Baroni, Silvia Molinelli, Chiara Paganelli

**Affiliations:** a Politecnico di Milano, Department of Electronics, Information and Bioengineering, Milano, Italy; b Centro Nazionale di Adroterapia Oncologica, Clinical Unit, Pavia, Italy; c RaySearch Laboratories, Eugeniavӓgen 18, 113 68, Stockholm, Sweden; d University of Pavia, Department of Clinical, Surgical, Diagnostic and Pediatric Sciences, Pavia, Italy

**Keywords:** Dose painting, Carbon ions, Sacral chordomas, Microstructural model, Diffusion MRI, TCP

## Abstract

**Background and purpose:**

Dose Painting (DP) in radiotherapy is a strategy to account for the tumor microstructural heterogeneity. This study evaluated a DP approach in carbon ion radiotherapy (CIRT), using cell count estimates derived from diffusion-weighted magnetic resonance imaging (DWI).

**Materials and methods:**

Thirty-seven large sacral chordoma (SC) patients were analysed. Voxel-wise cell count was estimated from DWI using a published microstructural model. A Poisson-based tumor control probability (TCP) model, fitted on 27 patients, guided DP optimization in a research version of RayStation 2024A. The approach was tested on 10 patients, comparing uniform-dose plans against two strategies: dose redistribution (DR), which maintained the mean gross tumor volume (GTV) dose, and dose escalation (DE), which allowed a 3 % increase. Plan evaluation on targets and organs at risk (OARs) included dose-volume histogram metrics (D_95%_, D_50%_, D_1%_) and dose-averaged linear energy transfer (LET_d_)-volume histogram metrics (L_98%_, L_50%_, L_1%_) to assess clinical acceptability. TCP gain quantified the benefit of biologically targeted strategies. TCP uncertainty was evaluated by propagating the microstructural model’s errors to generate best- and worst-case cell count maps.

**Results:**

DE and DR plans met clinical acceptability criteria. DE increased TCP from 75.5 ± 5.6 % to 83.3 ± 3.9 % (p < 0.001), with −3 to +5 percentage points variation under uncertainty. DR plans showed a TCP gain of 1.8 ± 1.0 percentage points. No significant dose or LET_d_ increase was observed in OARs, while DE plans showed a lower L_98%_ in GTV.

**Conclusions:**

Dose painting based on microstructural modelling in CIRT showed potential to improve TCP while sparing OARs.

## Highlights

• Diffusion-weighted imaging enabled dose painting in carbon ion radiotherapy.
• Average tumor control probability increased by 7.8 pp, ensuring organ sparing.
• Plans were robust to cell count uncertainties, limiting variations in tumor control.
• Linear energy transfer optimization was less effective in biologically targeted plans.

## Graphical abstract

**Figure:**
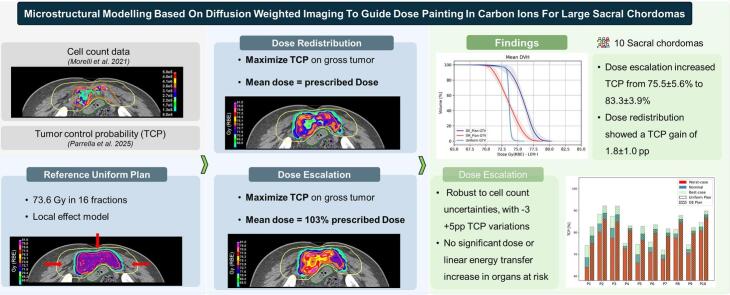

## 1 Introduction

In the last decade, biologically-targeted treatments have gained relevance in particle therapy [1], [2]. The high precision of pencil-beam scanning has enabled the investigation of dose-painting strategies, aiming to enhance therapeutic effectiveness. In this context, biologically-targeted approaches hold considerable clinical potential, particularly for treating radioresistant tumors such as sacral chordomas (SC). High linear energy transfer (LET) radiations, such as in carbon ion radiotherapy (CIRT) are especially suitable due to their enhanced relative biological effectiveness (RBE) and lower oxygen enhancement ratio (OER) [3]. Despite the radiobiological advantages and spatial precision of CIRT, large SCs may exhibit suboptimal dose and LET distributions [4], with evidence for a significantly lower dose-averaged LET (LET_d_) within the gross tumor volume (GTV) of locally relapsed cases [4], [5], [6], [7]. In response, new approaches have been explored to increase LET_d_ in potentially hypoxic regions [8], [9], [10]. However, optimizing LET_d_ at the voxel level remains challenging without spatially-resolved information identifying radioresistant subregions. For the same reason, the conventional dose delivery in radiotherapy follows a conservative strategy, where the RBE-weighted dose (D_RBE_) is uniformly delivered over the clinical target volume (CTV). In this regard, quantitative imaging modalities such as positron emission tomography and magnetic resonance imaging (MRI) are gaining relevance to derive biomarkers of tumor proliferation, hypoxia or surrogates of cell density [11], [12], [13], [14] and guide dose-painting strategies [13], [15].

Recent studies have explored the use of diffusion-weighted MRI (DWI) for non-invasive microstructural characterization of tumoral tissue, potentially addressing both inter- and intra-patient variability [16]. Various diffusion models were developed to capture the microstructural and functional properties of tumors. The apparent diffusion coefficient (ADC) is clinically used as a surrogate for cell density but lacks compartmental detail [17]. Intravoxel incoherent motion and restriction spectrum imaging capture additional features like microcirculation, at the cost of longer scans [18], [19]. In previous works, we developed a model to estimate microstructural parameters such as cell radius, density and tissues diffusivity from clinical DWI, using synthetic cellular substrates and Monte Carlo simulations of the diffusion signal [20], [21]. These parameters effectively distinguished patients based on cell proliferation levels [20], posing a solid base for biologically-targeted treatments. As such, the present work focuses on dose-painting strategies driven by tumor cell count estimates derived from DWI as proposed by Morelli et al. [20], while considering LET_d_ boosting on the GTV as a complementary component of the treatment.

When considering image-to-dose mapping, tumor control probability (TCP) models play a central role [22]. Different TCP models integrating biological factors from quantitative imaging have been proposed to guide dose escalation (DE) and redistribution (DR) strategies, both for intensity-modulated radiotherapy and particle therapy [23], [24], [25], [26], [27], [28]. Specifically, DR approaches aim at spatially redistributing the dose within the tumor, increasing it in potentially high-risk regions, while preserving the overall prescription dose. In contrast, DE strategies aim to selectively increase the dose delivered to high-risk subvolumes, enhancing local control without exceeding normal tissue constraints [15]. Given the absence of a TCP model able to discriminate controlled and relapsed SC cases, a revised version of the linear-quadratic model, fitted on patients’ cell count estimates, is hereby used to guide CIRT dose painting optimizations [29].

This study investigated the feasibility of dose painting strategies driven by an advanced DWI-based tumor microstructural model [20]. The proposed approach was integrated into an optimization framework that accounted for organs at risk (OARs) sparing, while evaluating the impact of uncertainties in cell density estimation. To our knowledge, this work presents the first integration of a DWI-based microstructural model into treatment optimization for CIRT, introducing a novel strategy that could enhance the effectiveness of biologically-targeted treatments.

## 2 Materials and methods

### 2.1 Data collection and elaboration

From a retrospective population including the first 50 patients diagnosed with non-metastatic SC, consecutively treated with CIRT at CNAO between 2013 and 2018, 37 patients were selected for this study. Inclusion and exclusion criteria and retrospective clinical data are summarized in Supplementary Tables S1 and S2. The study was approved by the local ethical committee (CNAO OSS 24/2021). Baseline data included planning CT scans (SOMATOM Sensation Open CT scanner, Siemens), T2-weighted MRI (T2w, Magnetom Verio, Siemens), DWI, and manually delineated structure contours provided by expert radiation oncologists on the treatment planning CT. CT were acquired with a resolution of 0.98 × 0.98 × 2 mm^3^, while DWI resolution ranged in 1.173–1.823 × 1.173–1.823 × 6 mm^3^. DWI and CT volumes were rigidly co-registered using T2w as an intermediate step, aiming to register the contours and extract microstructural information.

### 2.2 Cell count estimates and tumor control probability

Using the microstructural model developed by Buizza and Morelli et al. [20], [21], we derived patient-specific cell count estimates through the identification, for each voxel, of the optimal match between a library of simulated ADC values over in-silico cellular packings and the patient’s ADC, derived from DWI acquisitions at baseline. In addition, we derived the voxel-wise root mean squared error (RMSE) between the patient's ADC and the simulated ADC, to provide an indirect measure of uncertainty in the cell count estimates. The cell count maps were resampled to the dose grid resolution and the inverse transformations were applied to translate them to the CT space.

(1) $${\mathrm{TCP}}_{i}=100e^{{-e}^{e\gamma -\frac{{EQD4.6}_{i}}{D_{50i}}\left(e\gamma -\ln\ln2\right)}}={100e}^{-S_{i}}$$

(2) $$D_{50i}=\delta \left(1+\varphi \ln c_{i}\right)$$

(3) TCPROI=∏iNTCPiviVROI=100e-SROI

Cell count estimates were integrated into a revised Poisson TCP model (Eqs. (1), (2), (3)), where a voxel-wise logarithmic dependence on clonogenic cell count $c_{i}$ allowed to discriminate between patients with local control and local relapse [29]. A clonogenic fraction of 1 % was assumed, yielding an average cell density of approximately $10^{7}$ cells/cm^3^, consistent with literature values [30], [31].

Model parameters, described in Supplementary Table S3, were optimized through a bounded least-squares using 27 of 37 patients, with 10 held out for testing. The resulting TCP of the region of interest (ROI) was computed as the volume-weighted product of voxel-wise TCPs, as following Eq. (3), where *N* is the number of voxels in the ROI and viVROI is the relative volume of a voxel compared to the ROI [32]. A description of the TCP model, built and tested over the GTV to reduce MRI artifacts and tissue heterogeneity effects, is provided in a dedicated publication [29].

A custom optimization function was implemented in a research version of RayStation 2024A (RaySearch Laboratories, Stockholm, Sweden) to minimize the overall survival fraction (S_ROI_, Eq. (3)) within the selected region of interest. The cell count map was imported into the TPS through scripting, ensuring a voxel-wise correspondence between the cell count and the dose grid. The optimization function was benchmarked on a cubic computational phantom as reported in Supplementary Material A, Figs. S1, S2.

### 2.3 Dose painting in patient cases

The feasibility of the dose painting approach was evaluated on the 10 patients held out from the model fitting. For each patient, two plans were compared: a reference plan with a uniform dose distribution across the CTV, reflecting the standard of care, and a DE plan, where the above mentioned TCP function was applied to guide dose painting. Due to variations in TPS used, OARs constraints, dose prescriptions, and beam arrangements (Supplementary Table S2), all plans were re-designed to align with the current institutional treatment protocol. The protocol consists of a prescribed dose (D_P_) of 73.6 Gy (RBE)^1^delivered with a sequential approach in 16 fractions (4.6 Gy (RBE) per fraction), with target shrinkage after 9 fractions, moving from the low-risk CTV (CTV_LD_) to the high-risk CTV (CTV_HD_). RBE was estimated with LEM-I model, with α/β = 2 Gy (α_x_ = 0.1 Gy^−1^, β_x_ = 0.05 Gy^−2^, Dt = 30 Gy, r = 5 μm), while LET_d_ distributions were computed using the trichrome fragment spectra modelling [33]. Beams arrangement consisted of two fixed opposing lateral beams and one vertical beam, with the patient laying prone. More details on plan optimization are reported in Supplementary Material B.

The robust optimization was performed on a 3x3x3 mm^3^ dose grid with the RayStation pencil beam dose engine v7.0, using the clinical structure sets. Robust optimization functions were applied to the CTV, bowel, sigma and rectum. The robustness included 21 different scenarios, combining 3 range (i.e. 0 %, ±3%) and 7 different setup (±3 mm along the principal axes, and zero-shift) uncertainties. All plans included a low-weighted minimum LET_d_ objective of 43 keV/µm to the GTV, as per recent clinical practice at the institution [8].

In case of a DE plan, a non-robust TCP objective was applied to the GTV, while the remaining CTV received a uniform dose, as per standard of care. The clonogenic fraction was set to 1 % and, based on preliminary calibration, the TCP function was set to unit weight in the optimization. Dose painting was allowed within a D_RBE_ range of 95 % to 110 % D_P_. To prevent dose saturation at the upper limit of 81  Gy (RBE) (i.e. 110 % of D_P_) and considering the absence of normal tissue complication probability models for CIRT in the pelvis, DE plans were optimized to achieve a GTV mean dose ($\overset{¯}{D_{\mathrm{GTV}}}$) of 103 % D_P_. The optimization followed equation 4 where *S_i_, d_i_* and *v_i_* are respectively the voxel specific survival, D_RBE_ and volume, resulting in a custom dose escalation plan. More details on the objectives and constraints of DE and uniform plans are collected in Supplementary Tables S4, S5.

(4) $$\begin{array}{c} \min_{d_{i}}\sum_{i\in \mathrm{GTV}}S_{i}\frac{v_{i}}{V_{\mathrm{GTV}}}\\ Subject\;to\;\overset{¯}{D_{\mathrm{GTV}}}=1.03∙D_{P} \end{array}$$

Uniform and DE plans were compared in terms of dose metrics from the cumulative dose-volume histogram (DVH), including the minimum dose to the 95 %, 50 % and 1 % (D_95%_, D_50%_ and D_1%_, respectively) of GTV, CTV_HD_-GTV and CTV_LD_-GTV and LET_d_-volume histogram (LVH), with LET_d_ at 98 %, 50 % and 1 % of the volume (L_98%_, L_50%_, L_1%_). The Mann–Whitney *U* test (α = 0.05) was used to assess statistically significant differences in non-normally distributed metrics, as determined by Shapiro’s normality test (α = 0.05). Plans were considered clinically acceptable if they met predetermined clinical goals for the targets and surrounding OARs, including bowel, rectum, sigma, nerves and skin (Supplementary Table S6, S7). Specifically, target coverage was required to achieve D_95%_ > 0.95 D_P_, with D_1%_ < 1.15 D_P_ allowed in the GTV and D_1%_ < 1.05 D_P_ in non-escalated regions.

Plan robustness to cell count uncertainty was evaluated by simulating best- and worst-case scenarios, where cell counts were adjusted according to the RMSE from ADC fitting, representing decreased or increased values relative to the nominal estimates. This was achieved using two Gaussian curves that mapped RMSE values to scaling weights, ranging from 1 to 0.5 (i.e. best-case) and from 1 to 1.5 (i.e. worst-case). The Gaussian functions, showed in Supplementary Fig. S3, had a zero mean and a standard deviation matching that of the overall RMSE distribution observed in the fitting dataset (n = 27). The resulting three cell count maps were then used to estimate the uncertainty range in TCP predictions, considering dose distributions optimized on the nominal cell estimates.

(5) $${\Delta TCP}_{i}={\mathrm{TCP}}_{\mathrm{DE}}^{i}-{\mathrm{TCP}}_{\mathrm{Uniform}}^{i}$$

TCP maps for each scenario were compared to the uniform plan, and voxel-wise variations were evaluated as defined in Eq. (5).

Specifically, this evaluation was performed within subregions defined by discretizing the clonogenic cell count maps into three ranges (low, medium, and high cells count per voxel) based on the 33rd and 66th percentiles of the population (n = 10), corresponding to 2.1 10^5^ and 3.8 10^5^ cells/voxel.

The proposed pipeline was similarly applied to investigate an alternative dose painting strategy, consisting in DR. To this end, we replicated the same methodological framework while optimizing the mean dose to the GTV to match the prescription of 73.6 Gy (RBE), and adapting the targets clinical goals (Supplementary Table S6).

## 3 Results

**Fig. 1:**
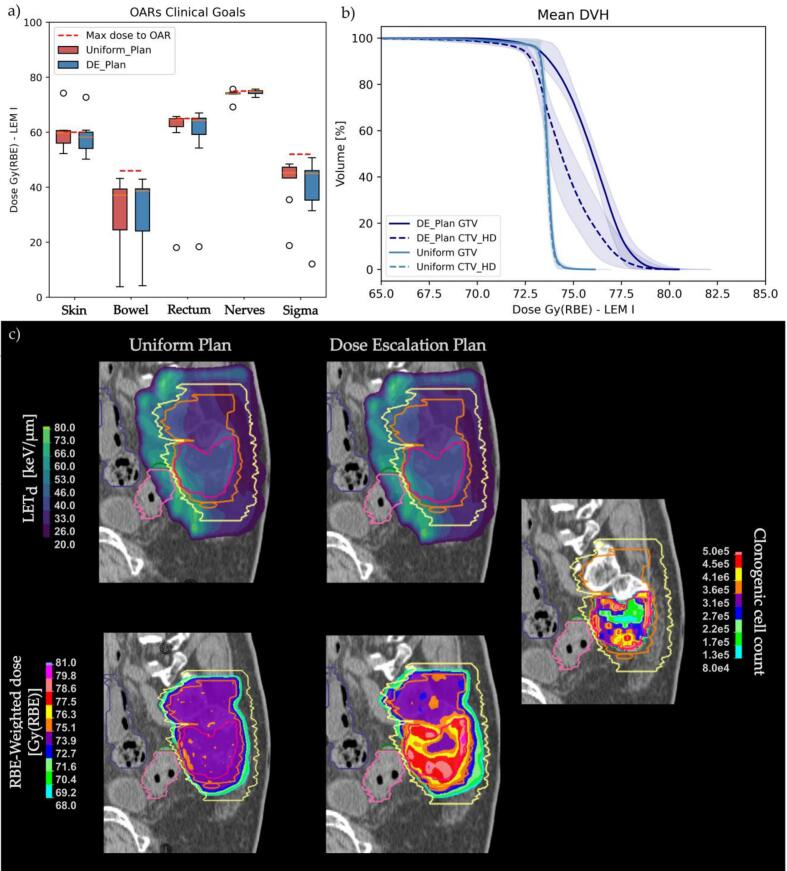
A) The average near-to-maximum dose as defined in clinical goals per OARs, and the corresponding limit, shown with a red dashed line. Boxplots contains 25th to 75th percentiles with whiskerks extending to 1.5 inter-quartile range (IQR). b) The mean GTV and CTV_HD_ DVH computed in uniform and DE plans. The bands show one standard deviation. c) Representative case of LET_d_ and dose distributions comparing Uniform and DE plans; on the right, the corresponding cell count distribution. (For interpretation of the references to colour in this figure legend, the reader is referred to the web version of this article.)

All uniform and DE plans met the clinical goals for both targets and OARs, as illustrated in Fig. 1a. One patient with a very large and superficial GTV (1270 cm^3^) did not meet skin clinical goals in either plan. A detailed summary of D_RBE_ and LET_d_ metrics for GTV and CTV_HD_-GTV is provided in Table 1 (Supplementary Table S8 for DR).

**Table 1:** RBE-weighted dose and LET_d_ metrics for Uniform and DE Plan, for GTV, CTV_HD_-GTV and CTV_LD_-GTV, expressed as median [25th–75th percentiles]. * represents a statistically significant difference between Uniform and DE Plan, following Mann-Whitney test, with alpha = 0.05.

		GTV		CTV_HD_-GTV		CTV_LD_-GTV	
		Uniform plan	DE plan	Uniform plan	DE plan	Uniform plan	DE plan
Gy (RBE)	D_95%_	73.2 [73.1–73.3]	73.3 [72.3–74.0]	73.1 [72.9–73.2]	72.2 [72.0–72.5]*	48.4 [44.0–54.3]	47.7 [44.0–52.4]
	D_50%_	73.6 [73.6–73.7]	76.0 [75.7–76.2]*	73.6 [73.6–73.7]	73.5 [73.5–73.6]	72.3 [70.9–72.8]	72.1 [71.6–72.4]
	D_1%_	74.5 [74.4–74.6]	79.5 [78.2–79.6]*	74.4 [74.2–74.5]	76.4 [76.2–76.6]*	74.2 [74.1–74.2]	75.8 [75.6–76.1]*
	$\overset{¯}{D}$	73.6 [73.6–73.6]	75.8 [75.8–75.9]*	73.5 [73.4–73.5]	73.5 [73.4–73.8]	67.7 [65.8–69.1]	67.2 [65.4–69.2]

keV/μm	L_98%_	42.8 [42.7–43.2]	41.5 [39.4–41.7]*	35.8 [32.9–39.6]	32.3[31.2–36.1]	30.6 [30.2–35.6]	29.5 [28.2–32.9]
	L_50%_	44.7 [44.1–45.2]	44.1 [42.9–45.8]	43.3 [42.9–44.3]	41.5 [41.3–43.9]	42.1 [40.2–44.2]	40.0 [39.1–43.3]
	L_1%_	53.0 [52.5–55.1]	55.6 [50.3–60.5]	57.0 [54.8–59.3]	60.4 [55.9–64.4]	64.5 [62.5–66.9]	66.6 [65.0–68.9]

DE plans achieved a median GTV D_95%_ of 73.3 Gy (RBE) with inter-quartile range (IQR) of [72.3–74.0] Gy (RBE), a D_1%_ of 79.5 [78.2–79.6] Gy (RBE), and a mean dose of 75.8 [75.8–75.9] Gy (RBE) in line with the study design (Fig. 1b). Concerning the CTV_HD_-GTV, which refers to the target volume outside the dose-escalated region, a slightly lower D_RBE, 95%_ and increased D_RBE,1%_ were noted. Similarly, CTV_LD_-GTV showed a higher D_1%_. Nonetheless, these differences remained within the clinical tolerance of 105 % D_P_.

The near-minimum LET_d_ (i.e. L_98%_) was slightly but significantly lower in the GTV of DE plans compared to uniform plans. No statistically significant differences were found in dose or LET_d_ metrics for the OARs, as shown in Supplementary Fig. S4. Representative examples of dose and LET_d_ distributions for uniform and DE plans are shown in Fig. 1c.

**Fig. 2:**
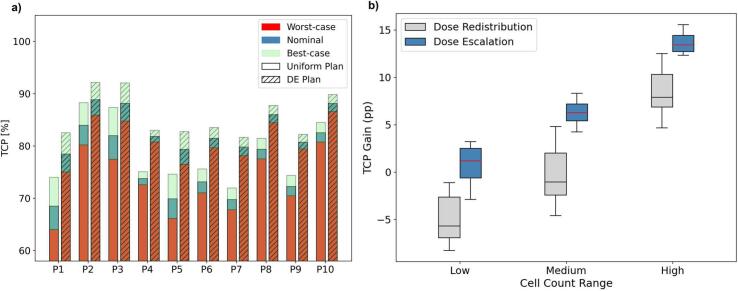
a) histograms display patient-specific TCP values computed over the GTV for the nominal, best-case, and worst-case scenarios, for both uniform and dose escalation (DE) plans. b) Boxplot displaying the population average TCP gain in percentage points (pp) computed as in Eq. (5), comparing dose redistribution (DR) and dose escalation (DE) strategies. Boxes shows the 25th and 75th percentiles, whiskers reach 1.5 inter-quartile range. Low, medium and high clonogenic cell count ranges were defined based on the 33rd and 66th percentiles of the population (n = 10).

The average TCP significantly increased from (75.5 ± 5.6) % for uniform plans to (83.3 ± 3.9) % for DE plans (p < 0.001), in the nominal cell count scenario. Patient-specific distribution of D_RBE_, TCP and clonogenic cell count are displayed in Supplementary Fig. S5. Accounting for cell count uncertainties resulted in an average TCP variation of −3 to +5 percentage points (pp) (Fig. 2a).

**Fig. 3:**
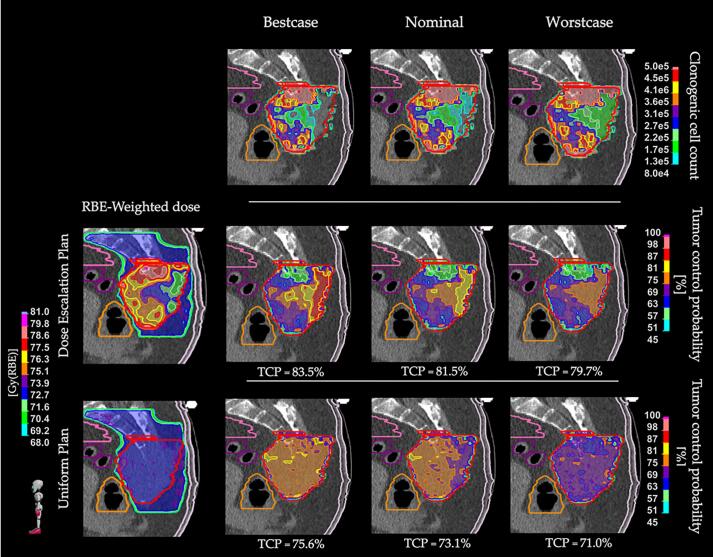
Sagittal view of a representative case illustrating TCP variations due to uncertainties in clonogenic cell count estimates (top row). The nominal cell count and corresponding TCP maps for the uniform (bottom row) and dose escalation (middle row) plans are shown. On the sides, the corresponding best-case (left) and worst-case (right) scenarios are displayed. All TCP maps were computed assuming the corresponding RBE-weighted dose distribution for uniform and dose escalation, shown on the left. The GTV is contoured in red, with the bowel in pink, rectum in orange, sigma in purple, and skin in white. The TCP values computed within the GTV for each scenario are reported below the maps. (For interpretation of the references to colour in this figure legend, the reader is referred to the web version of this article.)

Fig. 3 shows TCP variations under uncertainties in clonogenic cell count estimates, reporting the D_RBE_ distribution, the corresponding cell count map, and the resulting voxel-wise TCP map, for each scenario. Voxels with high cell densities experienced greater ΔTCP if compared to medium and low ranges, as displayed in Fig. 2b. Specifically, ΔTCP in highly dense regions was on average 13 percentage points (pp) higher than in regions with low cell count, both for DR and DE. Notably, DR showed an average ΔTCP of −5.0 pp in low cell count regions to enable TCP enhancement in high cell count subvolumes, with (+8.4 ± 2.3) pp. Mean DVHs for DE and DR are provided in Supplementary Fig. S6.

## 4 Discussion

This study investigated the feasibility and potential benefits of biologically-targeted dose escalation in CIRT by integrating advanced DWI-based tumor microstructural modelling into treatment planning. The proposed strategy enabled dose escalation in regions with high clonogenic cell count in the GTV, while enhancing LET_d_ and maintaining clinical acceptability.

The proposed TCP-guided DE plans allowed D_RBE_ to range between 95 % and 110 % of D_P_, with a +3 % increase of mean GTV dose. An increased dose was observed in CTV_HD_-GTV and CTV_LD_-GTV, but confined within the 105 % margin. Similar trends have been reported in dose painting studies and reflect the spatial pattern of the observed biological parameter (e.g. cell count), highlighting the need for precise localization of aggressive regions [24], [28]. D_RBE_ and LET_d_ metrics did not show significative differences between DE and uniform plans on OARs, while the GTV near to minimum LET_d_ was slightly but significatively lower in DE plans compared to uniform plans, suggesting that LET_d_ may warrant further optimization in biologically-targeted plans.

The DE strategy yielded significative improvements in TCP, with an average increase of 7.8 pp over the population. OARs acceptability criteria were met in all but one case with an exceptionally large and superficial tumor. Notably, similar limitations were observed in the clinically delivered plan, highlighting the intrinsic challenge of treating such large volumes, independently of the planning strategy. The observed TCP gain for DE plans aligned with previous findings on dose painting strategies, which reported improved probability of local control relative to uniform-dose approaches [14], [24]. Differently, in DR plans, the limited TCP gain of (1.8 ± 1.0) pp reflected the trade-offs between low and high cell count regions. This suggests that, when transitioning to dose painting approaches, a higher degree of dose heterogeneity within the target should be considered, allowing dose escalation in high-risk regions. Conversely, de-escalation in low-cellularity subvolumes should be avoided in the absence of supporting clinical or biological evidence.

To explore the impact of uncertainty in cell count estimates, we derived best- and worst-case maps by scaling nominal cell values according to the RMSE from ADC fitting. TCP evaluations under these conditions revealed a variation between +5 pp and −3 pp relative to the nominal case, consistent with previous reports of TCP reductions under input uncertainty [28]. These findings suggest that the proposed approach may retain biological benefits even when accounting for modelling uncertainties. Although it serves as a proof-of-concept, this investigation supports the development of biologically robust optimization in future planning frameworks, while highlighting the need for systematic methods to quantify biological uncertainty, which is essential to enhance robustness in a clinical context [22], [28].

Despite these promising results, some limitations must be acknowledged. First, the microstructural model did not differentiate between clonogenic and non-clonogenic cells, and for consistency with the literature, we adopted a fixed 1 % clonogenic fraction. While this assumption does not invalidate our conclusions, future improvements of the model are required. Moreover, cell estimates were derived from baseline DWI, without accounting for potential intra-treatment changes. Longitudinal MRI acquisitions could represent a valuable direction for improving both robustness and biological accuracy [34]. Additionally, the TCP model did not explicitly account for LET_d_ in the probability estimation, although LET-related effects are implicitly captured through RBE modelling. Implementing alternative models that incorporate LET_d_-dependent cell survival could better reflect the complexity of biological responses and support combined dose and LET_d_ painting strategies [35]. Dose painting with heavy ions has mostly focused to hypoxia targeting [14], [25], [27]. Conversely, the presented dose-painting approach was driven by cellular count. While the TCP performance in predicting local control and local relapse supports DE in regions with high clonogenic cell counts, further studies are required to validate this assumption and integrate additional markers of radioresistance, such as hypoxia.

Despite these limitations, our results support the feasibility of advanced microstructural modelling to guide biologically-targeted DE for large SC undergoing CIRT. The proposed DE strategy achieved significative TCP improvements while adhering to clinical requirements, and demonstrated potential robustness against uncertainties in cell count estimates. Although recalculations using alternative RBE models, such as the modified microdosimetric kinetic model, could provide additional insight, a direct comparison with LEM-based plans is challenging due to heterogeneous dose patterns and the incompatibility with the current TCP formulation. Future investigations should explore the robustness of dose painting across different RBE frameworks, combining multi-model RBE optimization with LET_d_ optimization to fully exploit the potential of biologically-targeted treatments. While validation in larger cohorts is required, this study introduces a novel DWI-based approach to dose painting in CIRT, showing promising clinical potential.

## CRediT authorship contribution statement

**Giovanni Parrella:** Conceptualization, Data curation, Formal analysis, Investigation, Methodology, Validation, Visualization, Writing – original draft. **Letizia Morelli:** Conceptualization, Data curation, Formal analysis, Methodology, Writing – review & editing. **Giuseppe Magro:** Conceptualization, Formal analysis, Methodology, Supervision, Writing – review & editing. **Lars Glimelius:** Conceptualization, Methodology, Software, Supervision, Writing – review & editing. **Jakob Ödén:** Conceptualization, Methodology, Software, Supervision, Writing – review & editing. **Mario Ciocca:** Data curation, Writing – review & editing. **Sara Imparato:** Data curation, Writing – review & editing. **Marco Rotondi:** Data curation, Writing – review & editing. **Maria Rosaria Fiore:** Data curation, Resources, Writing – review & editing. **Ester Orlandi:** Project administration, Resources, Writing – review & editing. **Guido Baroni:** Funding acquisition, Project administration, Resources, Supervision. **Silvia Molinelli:** Conceptualization, Formal analysis, Methodology, Supervision, Validation, Writing – review & editing. **Chiara Paganelli:** Conceptualization, Resources, Supervision, Writing – review & editing.

## Declaration of competing interest

The authors declare the following financial interests/personal relationships which may be considered as potential competing interests: Lars Glimelius and Jakob Ödén are employed at RaySearch Laboratories. This had no influence on the outcome or design of the study.

## Footnotes

1 with this nomenclature we refer to the ICRU93 definition
