# Supplementary material for: Microstructural modelling based on diffusion weighted imaging to guide dose painting in carbon ions for large sacral chordomas
Source: Phys Imaging Radiat Oncol. 2025 Dec 7;36:100887. doi: 10.1016/j.phro.2025.100887 (PMC12756709; doi:10.1016/j.phro.2025.100887)
Supplement: Supplementary Data 1 [file mmc1.pdf]

Microstructural Modelling Based On Diffusion Weighted Imaging To Guide Dose Painting In Carbon Ions For Large Sacral Chordomas

Supplementary materials

Table S1 Inclusion and exclusion criteria. Of the previously selected cohort of 50 SC patients, adhering to inclusion/exclusion criteria as in this table, 37 patients satisfied the criteria relative to the availability of complete clinical and dosimetric data, including baseline diffusion-weighted imaging (DWI) with b-values of 50, 400, and 1000 s/mm^2.

Inclusion criteria	Exclusion criteria
• Treatment with carbon ion radiotherapy delivering either 70.4 Gy (RBE) or 73.6 Gy (RBE) using LEM-I prescription in 16 fractions, with a sequential boost scheme including target shrinkage after 9 fractions<br>• Any degree of surgical resection (including biopsy or macroscopical resection)<br>• A minimum follow-up duration of 12 months;	• Metastatic disease<br>• No histological diagnosis<br>• Previous radiotherapy in affected region<br>• Concomitant chemotherapy<br>• Extensive metal instrumentation/implants<br>• Inability to hold the treatment positioning<br>• Pregnancy

Table S2 Clinical and demographic data

	**Relapsed**	**Control**
Number of patients	12	25
Sex assigned at birth	4/8 (Male/Female)	17/8 (Male/Female)
Number of fields	3 (4) – 2 (8)	3 (9) – 2 (16)
Dose per fraction [Gy (RBE)]	4.4 (12) – 4.6 (0)	4.4 (16) – 4.6 (9)
Treatment planning system	SyngoRT, Siemens (8) – RayStation, RaySearch (4)	SyngoRT, Siemens (13) – RayStation, RaySearch (12)
	Median (range)	*Median (range)*
Follow-up time [months]	-	39 (12-75)
Time-to-recurrence [months]	34 (17-64)	-

Table S3 Models' fitting parameters. Further details are discussed in a separated publication [28].

		γ	φ	δ [Gy (RBE)]
TCP_{LOG}	DWI-cell	3.39	41.45	0.13

Section A - Validation of TCP optimization on cubic phantom

We used a cubic phantom of 7x7x7 cm^3 with three distinct levels of cell count (Figure S1), specifically 1e6, 5e6, and 1e7 cells, to validate the correct behaviour of the TCP function implemented within the TPS. An ideal dose was computed within the contours of the target phantom, using a trust-region optimization approach implemented in SciPy, considering a clonogenic fraction of 1%. The bounded optimization problem limited the dose range between 68 Gy(RBE) and 78 Gy(RBE), while a composite objective function was defined to minimize overall survival, as implemented in the TPS, and to ensure an average dose close to the prescribed dose of 73.6 Gy. This analytical reference dose provided a benchmark to evaluate the dose optimized under similar conditions using 3 coplanar beams at 0° and ±90° on the TPS.

A graphical comparison of the cell count map, the reference ideal dose and the optimized dose, together with a profile of the maps along a line are shown in Figure S1. The comparison was evaluated in terms of Mean Absolute Error (MAE), which measures the average absolute difference between the optimized dose and the ideal dose and the Root Mean Squared Error (RMSE), evaluated within the target contours. Specifically, the MAE was 0.314 Gy, with a RMSE of 0.402 Gy. The distribution of the voxel-wise error is shown in Figure S2, together with a dose difference map and a scatter plot showing the relationship between the dose optimized in the TPS and the computed ideal dose.

These results demonstrate a strong agreement between the TPS-optimized dose and the ideal dose distribution, providing a robust foundation for the application of this optimization approach in patient cases.

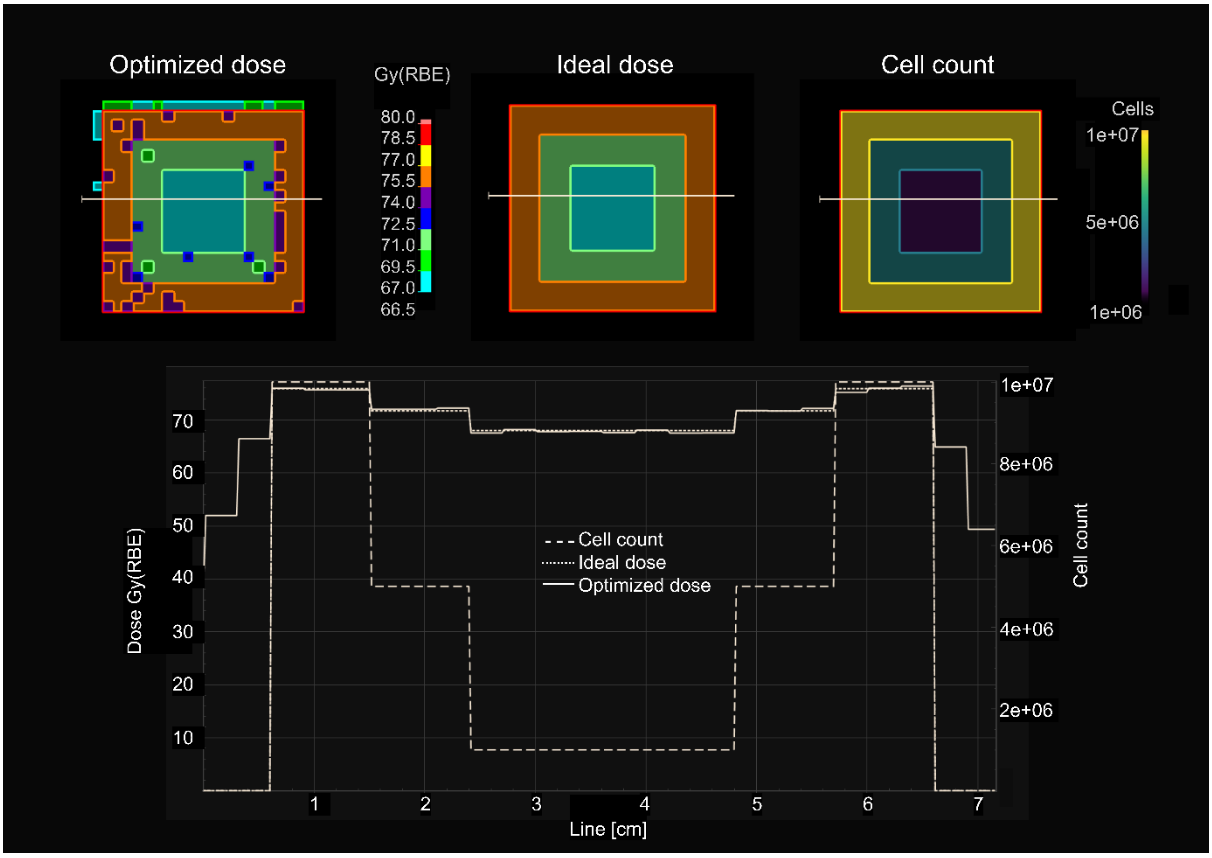

Figure S1 Graphical comparison of the optimized dose (Top left), the reference ideal dose (Top centre) and cell count map (Top right), on a central slice. Note that the clonogenic fraction scaling is not applied here. A profile of the maps along the white line are overlapped below, describing a good agreement between optimized and ideal dose.

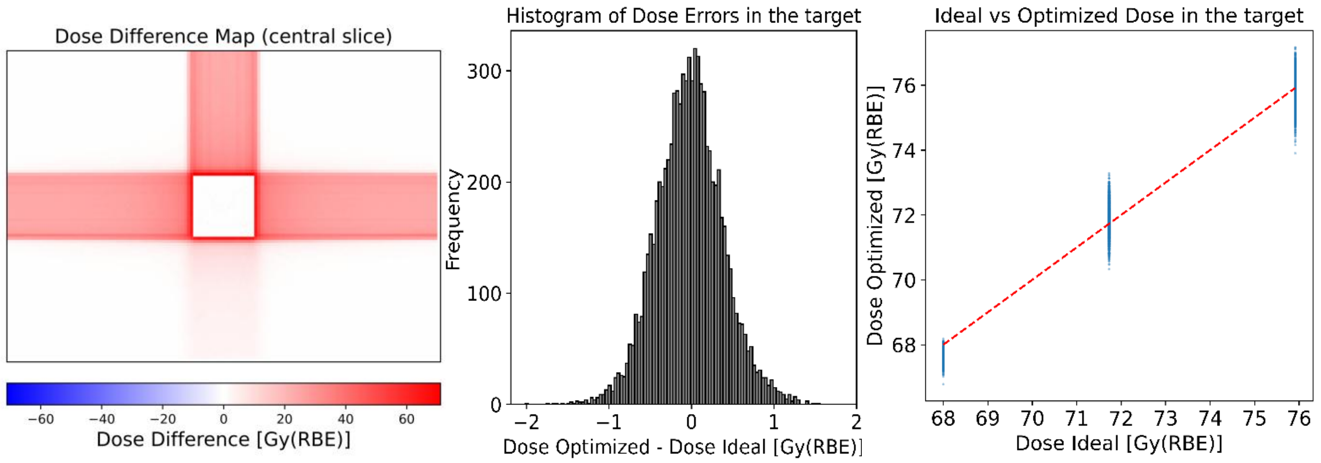

Figure S2 Central slice of dose difference map (Left), histogram of dose differences in the target (Centre) and scatter plot showing the relationship between the dose optimized in the TPS and the ideal dose. The red dashed line indicates the good agreement between the ideal and optimized doses, and the spread of the points around this line describes the degree of deviation.

Section B - Plan optimization and clinical goal

According to the clinical practice, the plans were optimized following a sequential boost approach, starting with 9 fractions to the CTV LD, followed by a target shrinkage to CTV HD in the remaining 7 fractions; a separate beam-set was created for each of two the treatment parts. All fractions were optimized with minLET$_d$ objectives on the GTV, set at 43 keV/µm.

Dose painting plans were characterized by the inclusion of the custom TCP optimization function on the GTV, aiming at minimizing tumour cell survival, combined with a mean-dose (EUD with a = 1) objective to keep the GTV mean dose consistent with the planning strategy (DR or DE). In this setting, regions of the CTVs not included in the GTV (i.e. CTV HD – GTV and CTV LD – GTV) were optimized following the same objectives as in the reference uniform-dose plan. Robust optimization functions were applied to the CTV, bowel, sigma and rectum. The robustness included 21 different scenarios, as a result of the combination of 3 range (i.e. 0%, ±3%) and 7 different setup (±3 mm along the principal axes, and zero-shift) uncertainties.

Relative objective weights were chosen empirically and adapted to each patient case to ensure adherence to clinical goals for OARs and target coverage (Tables S6–S7) while respecting the intended planning strategy. The TCP objective was assigned unit weight, corresponding to roughly 0.5% of the weight used for uniform CTV–GTV dose objectives. A systematic sensitivity analysis of all weights was not performed in this feasibility study and is left for future work. Objectives were linearly scaled between the 9- and 7-fraction beam sets. Below, the details on the objective and constraints used.

Table S4 Objectives and constraints applied to the targets, used as a starting point for the optimization of dose escalation plans. Note that the CTV LD is only treated for 9 fractions. In the other cases, the values refers to the entire treatment. * represent a robust optimization.

ROI	Objectives	Constraints
GTV *(9+7 fractions)*	▪ TCP function<br>▪ minLETd 43 keV/µm<br>▪ maxEUD (a=1) 7581 cGy(RBE)	▪ Max dose 8100 cGy(RBE)<br>▪ Min dose 6991 cGy(RBE)
CTV HD – GTV *(9+7 fractions)*	▪ *minDVH 7140 cGy(RBE) at 97% vol.<br>▪ Uniform dose 7360 cGy(RBE)	▪ Max dose 7640 cGy
CTV LD – GTV *(9 fractions)*	▪ *minDVH 4000 cGy(RBE) at 97% vol.<br>▪ Uniform dose 4140 cGy(RBE)	▪ Max dose 4300 cGy

Table S5 Objectives and constraints applied to the targets, used as a starting point for the optimization of Uniform-dose plans. Note that the CTV LD is only treated for 9 fractions. In the other cases, the values refers to the entire treatment. * represent a robust optimization.

ROI	Objectives	Constraints
GTV *(9+7 fractions)*	▪ minLETd 43 keV/µm	-
CTV HD *(9+7 fractions)*	▪ *minDVH 7140 cGy(RBE) at 97% vol.<br>▪ Uniform dose 7360 cGy(RBE)	▪ Max dose 7640 cGy
CTV LD *(9 fractions)*	▪ *minDVH 4000 cGy(RBE) at 97% vol.<br>▪ Uniform dose 4140 cGy(RBE)	▪ Max dose 4300 cGy

Table S6 Clinical Goals on targets for the different treatment plans investigated. Dp = 73.6 Gy(RBE). Note that for DE and DR plans, the metrics are computed over CTV_HD excluding the GTV, in order to assess the coverage of the region of CTV not covered by dose painting or dose redistribution

ROI		Uniform Plan	DE Plan	DR Plan
GTV	At least	$D_{95\%} > 0.95Dp$		
	At most	$D_{1\%} < 1.05Dp$	$D_{1\%} < 1.15Dp$	$D_{1\%} < 1.07Dp$
CTV_{HD} or CTV_{HD}-GTV	At least	$D_{95\%} > 0.95Dp$		
	At most	$D_{1\%} < 1.05Dp$	$D_{1\%} < 1.05Dp$	$D_{1\%} < 1.05Dp$

Table S7 Clinical Goals on OARs. Dp = 73.6 Gy(RBE). Note that the clinical goals on some ROI are defined over absolute volume (cm^3).

ROI	Clinical Goal	Notation
Bowel	At most 46 Gy(RBE) to 1cm^3	**D_{1cc} < 46 Gy(RBE)**
Rectum	At most 65 Gy(RBE) to 1 cm^3	**D_{1cc} < 65 Gy(RBE)**
Sigma	At most 52 Gy(RBE) to 1 cm^3	**D_{1cc} < 52 Gy(RBE)**
Skin	At most 60 Gy(RBE) to 5 cm^2	**D_{5sc} < 60 Gy(RBE)**
Nerves	At most 75 Gy(RBE) to 1%	**$D_{1\%}$ < 75 Gy(RBE)**

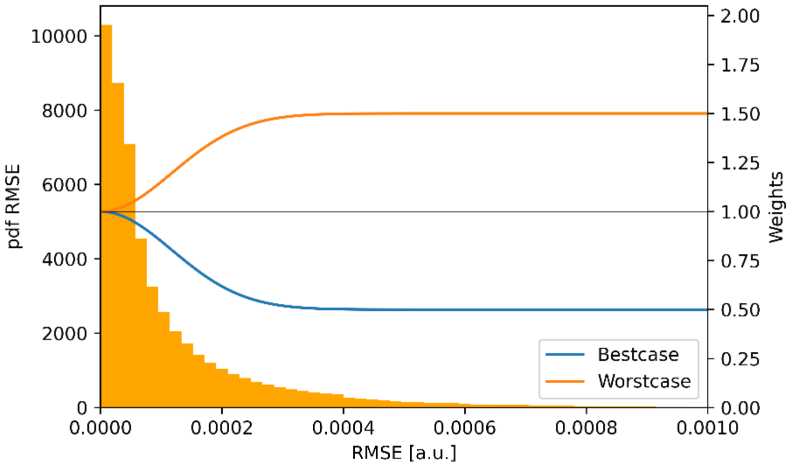

Figure S3 Representation of the Gaussian curves (i.e. Best-case in blue, Worst-case in orange) that convert RMSE in scaling weights to address cell count uncertainty. In orange on the background the histogram of the RMSE distribution over the TCP fitting dataset (n=27). The curves have zero mean and a standard deviation matching that of the RMSE distribution observed in the fitting dataset (i.e. 0.000117).

Table S8 RBE-weighted dose and LET_d metrics for DR Plan, for GTV, CTV_{HD}-GTV and CTV_{LD}-GTV, expressed as median [25th-75th percentiles]. * represents a statistically significant difference between Uniform and DR Plan, following Mann-Whitney test, with alpha = 0.05.

		GTV	CTV_{HD}-GTV	CTV_{LD}-GTV
Gy(RBE)	$D_{RBE,95\%}$	70.9 [70.5–71.3]*	72.0 [71.5–72.2]*	47.7 [44.0–52.9]
	$D_{RBE,50\%}$	73.5 [73.5–73.7]	73.4 [73.4–73.5]*	72.0 [71.6–72.1]
	$D_{RBE,1\%}$	77.4 [77.1–77.6]	76.1 [75.8–76.3]*	75.3 [75.2–75.7]*
	$\bar{D}$	73.7 [73.6–73.7]	73.4 [73.3–73.5]	67.0 [65.5–68.9]
keV/μm	$L_{98\%}$	40.7 [39.5–41.6]*	32.5 [31.4–36.1]	29.5 [28.4–32.5]
	$L_{50\%}$	43.7 [42.9–44.8]	41.7 [41.1–43.0]	40.4 [39.2–42.6]
	$L_{1\%}$	52.5 [51.1–57.2]	57.9 [55.0–61.5]	65.6 [61.9–67.9]

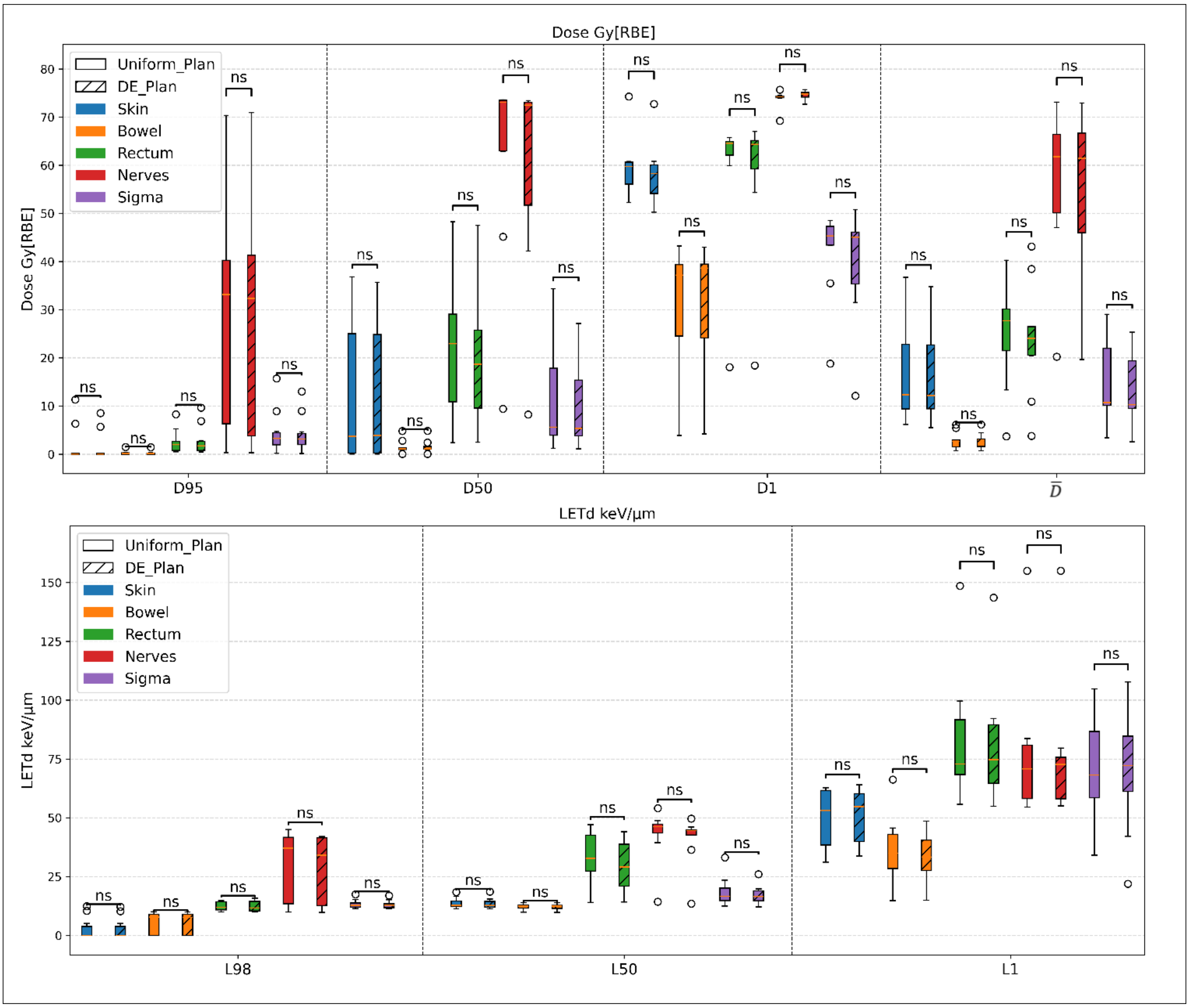

Figure S4 Boxplot of the patients' averages of the computed metrics for RBE-weighted dose and LET_d for the main OARs. Ns = not significative

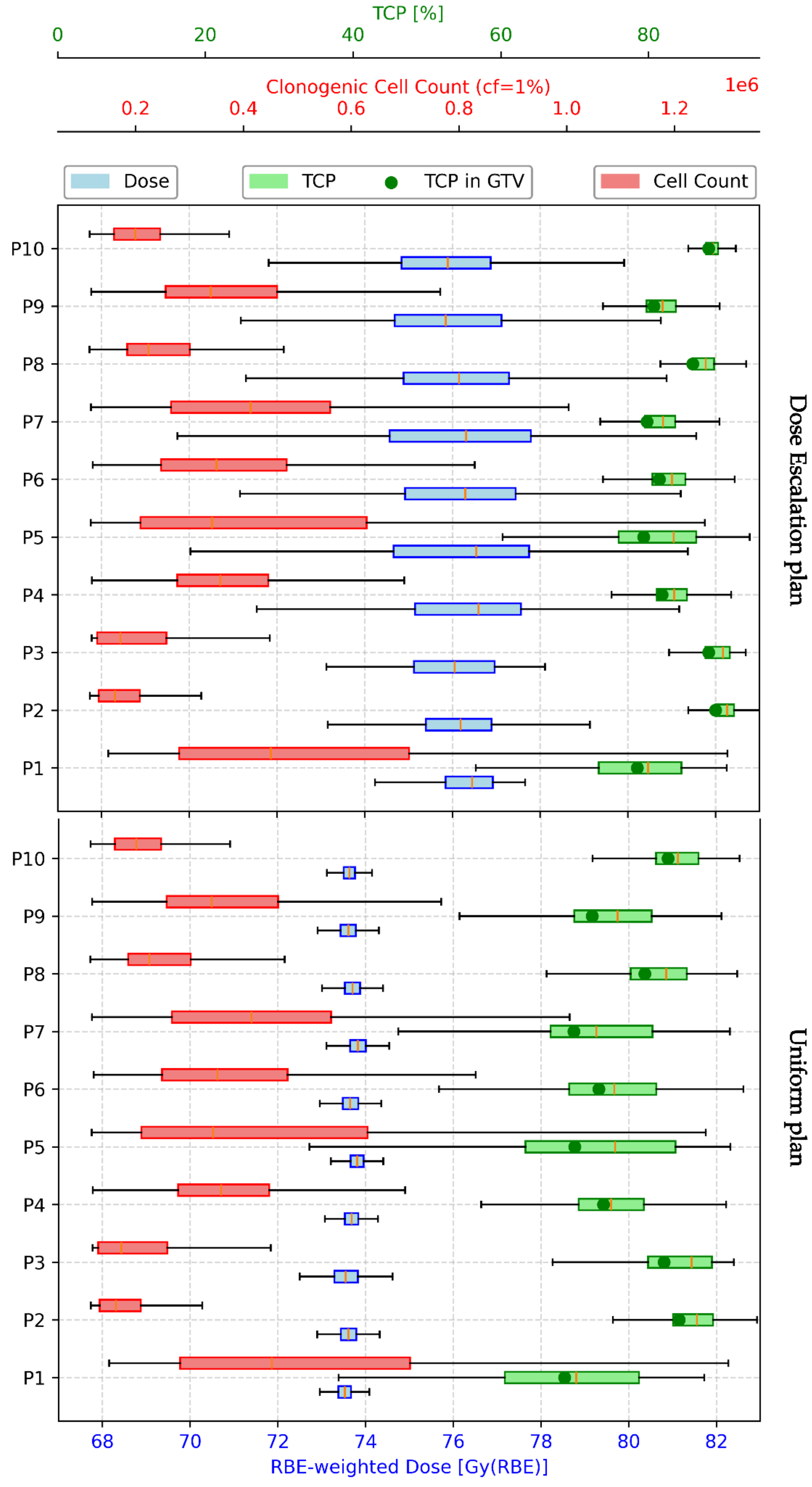

Figure S5 Boxplot comparing clonogenic cell count (Red), RBE-weighted dose (Blue), voxel-wise TCP (Green) and patients' TCP (Scatter green) for each case, comparing Uniform Plan (Bottom) and dose escalation plan (Top).

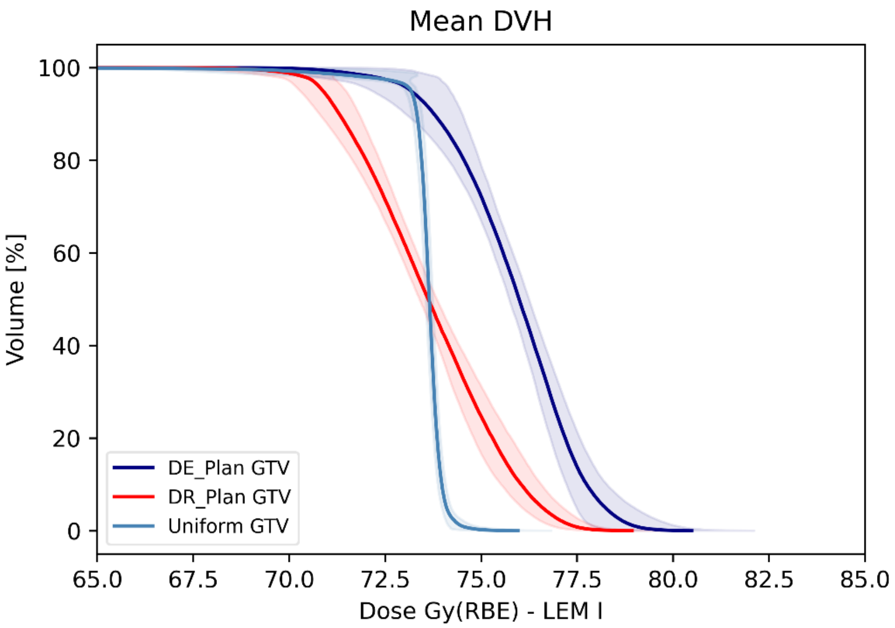

Figure S6 Mean DVH for dose escalation (DP Plan) and dose redistribution (DR Plan), compared to the uniform dose plan, averaged over 10 patients. The bands show 1 standard deviation.
